# Circulating Tumor DNA-Guided Adjuvant and Post-Adjuvant Therapy in Resected Colorectal Cancer: From Molecular Residual Disease Detection to Proven Clinical Utility

**DOI:** 10.3390/biomedicines14081674

**Published:** 2026-07-25

**Authors:** Thai Hau Koo, Rishi Chowdhary, Kirti Arora, Kelly Chun Lynn Lai, Andee Dzulkarnaen Zakaria

**Affiliations:** 1 Department of Internal Medicine, Cleveland Clinic Akron General, Akron, OH 44307, USA; koot@ccf.org (T.H.K.); arorak3@ccf.org (K.A.); 2 Division of Gastroenterology and Hepatology, MetroHealth Medical Center, Case Western Reserve University, Cleveland, OH 44109, USA; 3 Division of Colorectal Cancer, Department of Surgery, School of Medical Sciences, Hospital Pakar Universiti Sains Malaysia, Kelantan 16150, Malaysia; 4 Department of Medicine-Pediatrics, MetroHealth Medical Center, Case Western Reserve University, Cleveland, OH 44109, USA; rchowdhary@metrohealth.org; 5 Department of Internal Medicine, Hospital Sungai Buloh, Taylor’s University School of Medicine, Sungai Buloh, Selangor 47000, Malaysia; kellylaichunlynn21@gmail.com

**Keywords:** circulating tumor DNA, colorectal cancer, adjuvant chemotherapy, liquid biopsy, DYNAMIC trial, ALTAIR

## Abstract

Colorectal cancer (CRC) kills approximately 850,000 people annually and is the second leading cause of cancer mortality worldwide. After curative-intent surgical resection, recurrence rates of 15–20% in stage II and 30–40% in stage III diseases highlight the fundamental limitations of pathological staging as the sole basis for adjuvant treatment decision-making. Circulating tumor DNA (ctDNA), which comprises tumor-derived cell-free DNA fragments shed into the bloodstream by dying cancer cells, offers a direct and dynamic readout of molecular residual disease (MRD) in the postoperative period. Because ctDNA clears within hours of macroscopically complete surgery, any persistently detectable postoperative signal reflects viable microscopic disease rather than surgical contamination. Over the past decade, evidence has matured rapidly from proof-of-concept observational studies to completed randomized clinical trials, culminating in the recent publication of DYNAMIC-III and ALTAIR in 2025–2026. The DYNAMIC trial established that ctDNA-guided management reduced adjuvant chemotherapy use from 27.9% to 15.3% in stage II colon cancer while maintaining an equivalent five-year recurrence-free survival (88% vs. 87%; difference 1.1%, 95% CI 5.8% to 8.0%). The GALAXY study confirmed ctDNA as the most powerful prognostic biomarker in resected CRC, with a disease-free survival hazard ratio of 11.99. Critically, DYNAMIC-III demonstrated that escalating chemotherapy intensity in ctDNA-positive stage III patients did not improve recurrence-free survival (HR 1.11, *p* = 0.6), and the phase III ALTAIR trial showed that trifluridine/tipiracil failed to significantly improve disease-free survival at the point of molecular recurrence (HR 0.79, *p* = 0.107). This review critically synthesizes the current evidence for ctDNA-guided treatment decisions in resected CRC across three therapeutic strategies: de-escalation in ctDNA-negative patients, escalation in ctDNA-positive patients, and post-adjuvant therapy at the point of molecular recurrence. Future progress requires biomarker-matched escalation strategies, adaptive trial designs, and formal validation of ctDNA clearance as a surrogate endpoint for predicting patient outcomes.

## 1. Introduction

Colorectal cancer remains the third most common malignancy and the second leading cause of cancer-related death globally, accounting for approximately 1.9 million new diagnoses and 850,000 deaths annually [1]. The majority of patients present with locoregional disease amenable to curative-intent resection; however, five-year recurrence rates, roughly 15–20% for stage II and 30–40% for stage III colon cancer, reveal a critical gap between surgical cure and biological cure [2]. For decades, adjuvant treatment decisions have rested on pathological stage, supplemented by mismatch repair (MMR) status, tumor grade, and a handful of high-risk clinicopathological features. While useful as population-level risk stratifiers, these tools cannot distinguish individual patients harboring viable micrometastatic deposits from those who are truly cured after surgery.

This imprecision can lead to two distinct clinical harms. On one hand, a substantial proportion of patients with stage II disease receive adjuvant fluoropyrimidine chemotherapy, which confers an absolute five-year survival benefit of only 3–5% at the cost of fatigue, cytopenias, mucositis, and neuropathy [3]. On the other hand, some patients with stage III disease relapse rapidly despite standard oxaliplatin-based chemotherapy, suggesting that their disease was chemoresistant from the outset and that earlier knowledge of that resistance might have redirected treatment toward more appropriate alternatives. Conventional biomarkers cannot fill either gap: carcinoembryonic antigen (CEA) levels rise only after macroscopic recurrence, cross-sectional imaging has a spatial resolution of 5–7 mm and cannot detect submillimeter deposits, and mutational profiling of RAS/BRAF/MMR status refines prognosis without reliably predicting adjuvant benefit at the individual patient level.

Circulating tumor DNA (ctDNA) has emerged as a fundamentally different class of biomarker. Tumor cells undergoing apoptosis and necrosis release fragmented genomic DNA into the bloodstream; this cell-free DNA carries tumor-specific somatic mutations, methylation patterns, and nucleosomal footprints that distinguish it from germline-derived cfDNA [4]. After curative resection, ctDNA is cleared from circulation within hours, reflecting the brief half-life of cfDNA fragments. Any postoperative ctDNA signal identifies patients with a persistent viable tumor burden below the threshold of conventional detection, a state now widely termed molecular residual disease (MRD). The clinical stakes are high: in the largest prospective dataset to date, ctDNA-positive patients had a disease-free survival hazard ratio exceeding 11 compared with ctDNA-negative patients, a magnitude unmatched by any prior prognostic biomarker in resected CRC [5,6].

The physical and molecular characteristics of the analyte itself underlie every downstream assay-performance argument. Cell-free DNA circulates predominantly as mononucleosomal fragments with a modal length of approximately 166 base pairs, corresponding to the roughly 147 base pairs of DNA wrapped around the histone octamer together with an adjacent linker segment, upon which a series of shorter subpeaks spaced at intervals of approximately 10 base pairs is superimposed and reflects nuclease cleavage of the exposed helical face [7]. Tumor-derived fragments deviate systematically from this germline pattern, being shifted toward shorter lengths with a relative enrichment below approximately 150 base pairs that arises from altered nucleosome positioning and more accessible chromatin in malignant cells, such that the nucleosomal footprint of ctDNA is measurably distinct from that of leukocyte-derived cfDNA [8,9]. This differential is not merely descriptive, because the shorter fragments carry a disproportionate share of the tumor signal, so that in silico or physical selection of sub-150-base-pair molecules enriches the mutant allele fraction and improves detection at the low tumor burdens characteristic of the postoperative setting [8]. Fragment length, end-motif composition, and nucleosome-occupancy signatures therefore constitute an information layer that is independent of, and complementary to, the somatic mutations tracked by conventional panels, and their exploitation is central to the assay strategies discussed below [10].

The translational journey from this compelling biology to actionable clinical guidance has not been linear or unambiguously positive. The proof-of-concept was established in 2016 when Tie et al. demonstrated that detectable ctDNA four weeks after stage II colon cancer resection predicted recurrence with specificity approaching 100%, anticipating radiographic recurrence by a median of several months [11]. The subsequent decade produced a series of prospective observational studies, real-world programs, and ultimately completed randomized trials. The results have forced a reckoning: ctDNA is extraordinary at predicting relapse, but it has not yet been proven that it can prevent relapse.

**Figure 1:**
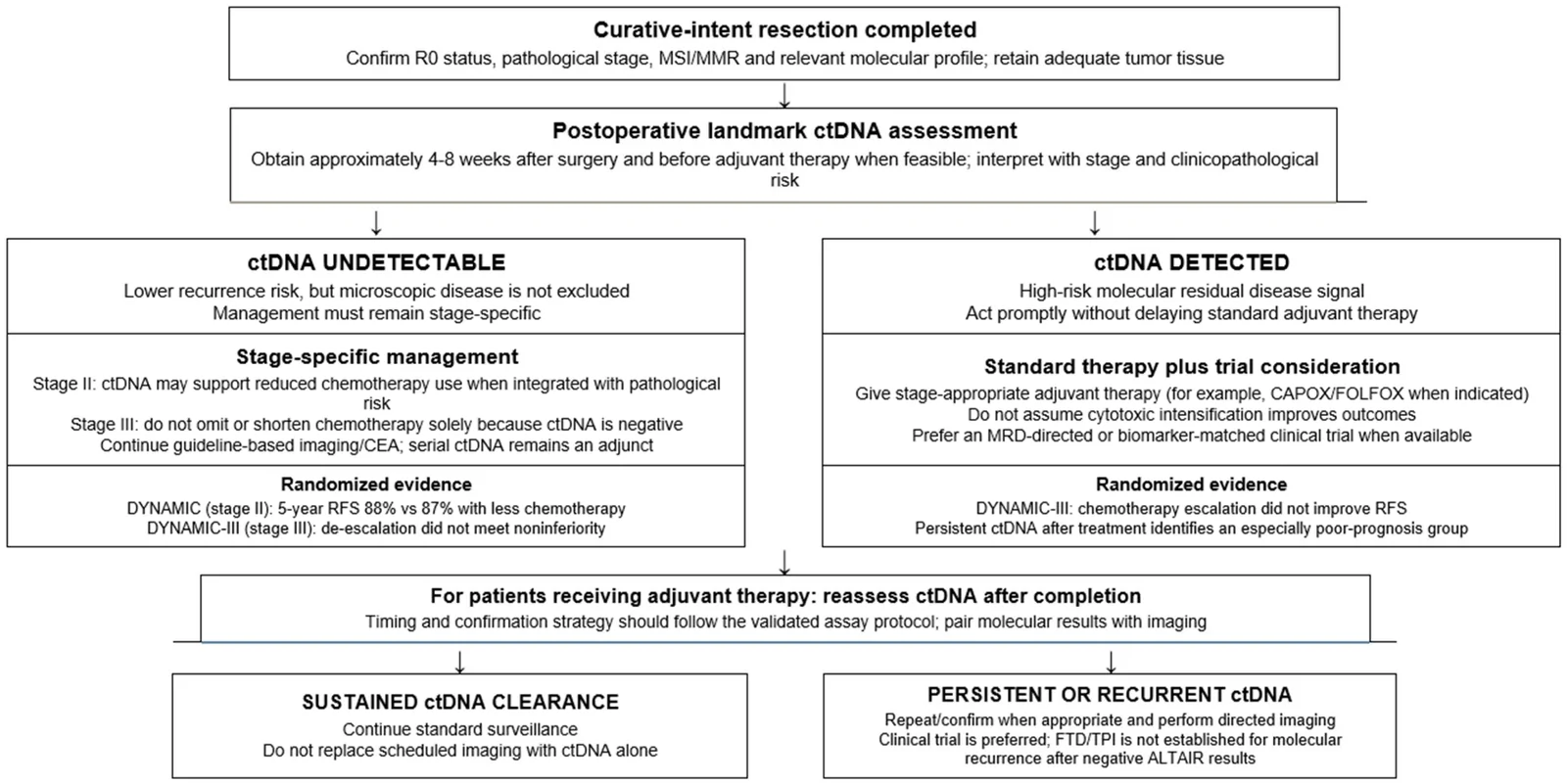
Postoperative ctDNA-guided clinical decision pathway in resected colorectal cancer. Schematic depicting the three major therapeutic strategies enabled by postoperative ctDNA testing: de-escalation in ctDNA-negative patients (left branch), standard adjuvant therapy with intensified surveillance in ctDNA-positive patients (middle), and trial-based escalation or treat-on-molecular-recurrence in confirmed high-burden MRD-positive patients (right branch). The optimal timing for the MRD landmark (4–7 weeks post-resection) and the current evidence basis for each pathway (DYNAMIC for de-escalation; DYNAMIC-III and ALTAIR for escalation and post-adjuvant TOMR) are indicated. Shaded boxes denote strategies with level 1 randomized evidence; unshaded boxes denote strategies requiring further prospective trial validation. ACT = adjuvant chemotherapy; TOMR = treat-on-molecular-recurrence; MTM/mL = mutant tumor molecules per milliliter.

This review provides a critical synthesis of the current evidence organized around the central clinical question: When does acting on ctDNA improve patient outcomes? We examine the biological and technical foundations of MRD detection, analyze randomized and prospective clinical trial data by disease stage and therapeutic strategy, dissect the mechanistic reasons for the persistent prognostic-to-predictive gap, and outline the scientific and regulatory requirements needed to close this gap. The recently published ALTAIR phase III trial and mature DYNAMIC-III readout together make this an opportune moment for such a reappraisal (Table 1). The proposed postoperative ctDNA-guided clinical decision pathway for resected CRC is summarized in Figure 1.

## 2. Biological and Technical Foundations of ctDNA-Based MRD Detection

### 2.1. Molecular Residual Disease: Definition and Biological Rationale

Molecular residual disease refers to the presence of viable tumor cells below the detection threshold of conventional staging and surveillance tools. After macroscopically complete resection, micrometastatic foci may reside in the liver, regional nodes, or distant sites in either a dormant or proliferating state. Their detection hinges on the release of ctDNA into the systemic circulation, a process proportional to cell death and, hence, to the tumor cell burden. The half-life of cfDNA in circulation has been estimated at approximately 1–2 h in most studies, meaning that plasma ctDNA concentrations reflect near-real-time tumor dynamics rather than cumulative damage [4]. This rapid clearance underpins the specificity of postoperative ctDNA: the persistent detection of tumor-specific DNA after surgery cannot be attributed to the primary tumor remnant once the resection specimen is confirmed as pathologically complete.

Tumor burden is the primary determinant of ctDNA concentration: stage IV metastatic disease may shed ctDNA, constituting 10–50% of total plasma cfDNA, while single micrometastatic clusters shed at variant allele fractions (VAF) of 0.001–0.01% or below [21]. This dynamic range of more than four orders of magnitude means that MRD assays must be analytically ultrasensitive while preserving specificity against the background noise of somatic mutations in non-tumor cells, particularly clonal hematopoiesis of indeterminate potential (CHIP).

Clonal hematopoiesis of indeterminate potential warrants specific consideration because it is the dominant source of false-positive plasma variants. Somatic mutations arising in hematopoietic stem cells, most frequently affecting DNMT3A, TET2, ASXL1, TP53, and PPM1D, are shed into plasma by leukocytes and are detectable in up to one-fifth of individuals older than 70 years, precisely the demographic in which colorectal cancer is most prevalent [22]. Several complementary strategies mitigate this interference. Sequencing of matched white blood cells at comparable depth allows variants shared between the leukocyte and plasma compartments to be subtracted, and remains the reference standard for unambiguous assignment of a plasma variant to tumor rather than hematopoietic origin [22]. Tumor-informed assays circumvent much of the problem by design, because the personalized panel tracks only variants pre-identified in the resected tumor and therefore does not report clonal-hematopoiesis mutations absent from that reference [23]. Where matched leukocyte sequencing is impractical, computational classifiers that distinguish hematopoietic from tumor-derived variants on the basis of sequence context and fragment features offer a plasma-only alternative, and error-corrected sequencing with unique molecular identifiers further suppresses technical noise near the detection threshold. Standardizing these filtering approaches across platforms remains an active research priority, since residual clonal-hematopoiesis signal degrades specificity most severely in the low-burden postoperative window where escalation decisions are contemplated.

### 2.2. Tumor-Informed Versus Tumor-Naive Assay Strategies

Two broad analytical strategies exist for detecting ctDNA in the postoperative context, each with distinct trade-offs. Tumor-informed (bespoke) assays first sequence the resected tumor to identify patient-specific somatic single-nucleotide variants and then design a personalized multiplex panel targeting 16–50 or more of those variants simultaneously in plasma. The Signatera assay (Natera, Austin, TX), which employs a 16-variant targeted next-generation sequencing panel with unique molecular barcoding, achieves a limit of detection of approximately 0.01% VAF and is the most extensively clinically validated tumor-informed platform in CRC, having been used in DYNAMIC, GALAXY, BESPOKE, and ALTAIR [23]. Ultra-deep hybrid-capture approaches can push analytical sensitivity toward 0.004–0.005% VAF but require substantially greater sequencing depth and cost.

Tumor-naive (tumor-agnostic) assays use a fixed panel of recurrently mutated genes or genome-wide epigenomic features and do not require tumor tissue, offering a faster turnaround and deployment without archival FFPE material. The Guardant LUNAR/Reveal platform and methylation-based systems, such as SPOT-MAS, represent this class [24]. Their analytical sensitivity per individual timepoint is generally lower (0.1–0.25% VAF for fixed mutation panels), but they offer two practical advantages: independence from tissue availability and the capacity to detect clonal evolution beyond the primary tumor. A subset analysis within GALAXY comparing tumor-informed Signatera with the tumor-naive xM assay combining methylation density and SNV detection demonstrated that the xM assay achieved a clinical sensitivity of 61.1% at the four-week landmark with a specificity of 87.9%, which showed lower absolute performance, but was clinically meaningful when paired with serial testing [6]. The two strategies are complementary rather than competitive (Table 2).

A recent advance illustrates how the tumor-agnostic category itself is evolving beyond fixed panels toward genome-scale interrogation of plasma. Martín-Arana and colleagues applied whole-exome sequencing directly to postoperative plasma in two independent cohorts of patients with localized colon cancer, an approach designated whole-exome tumor-agnostic sequencing, and reported a molecular-residual-disease sensitivity of 86.7% and 100.0% in the discovery and validation cohorts, respectively, at a specificity of 95.0% when a single detected variant was treated as indicative of positivity [25]. This performance exceeded that of both personalized tumor-informed assays and custom tumor-agnostic panels applied to the same samples, and a cost-reduced derivative that tracked the sixteen highest-variant-allele-fraction alterations identified in baseline plasma reproduced the sensitivity of the full-exome approach [25]. The broader coverage afforded by exome-scale plasma sequencing also captured clinically actionable alterations and evidence of clonal evolution that narrow tumor-informed panels would have missed, a point developed further in Section 4.7. These findings position plasma whole-exome sequencing as a promising route to mitigate the false-negative problem that constrains current landmark assays, although prospective validation in larger cohorts and formal cost-effectiveness analysis remain necessary before clinical adoption [26].

### 2.3. Signal Modalities: Somatic Mutations, Methylation, and Fragmentomics

Three orthogonal molecular layers can be interrogated from cfDNA, and their integration represents an active frontier. Somatic single-nucleotide variants and short insertions or deletions provide the specificity backbone of most current MRD assays because tumor-specific variants are not present in germline DNA [27]. Tumor-specific CpG methylation patterns differ profoundly between cancer and normal tissues and are detectable in cfDNA with sensitivity independent of mutational burden; in a prospective study of 1122 patients across CRC stages I–III, methylation-based ctDNA detection identified MRD in 56% of samples where mutation tracking was negative and independently predicted recurrence [28]. Fragmentomic features include the characteristic shortening of tumor-derived cfDNA fragments (145 bp vs. 166 bp for non-tumor cfDNA), nucleosome positioning signatures, and 5′ end-motif distributions, provide a mutation-independent third signal particularly valuable in tumors with few trackable variants [10]. Multimodal platforms combining methylation density with fragment-size profiling have demonstrated area under the curve values approaching 0.96–0.99 for CRC detection in preliminary series, though clinical MRD validation data remain limited and prospective trials using multimodal endpoints are needed. Integrating these orthogonal layers is also emerging as a strategy to address the sensitivity ceiling of single-analyte ctDNA in advanced and treatment-refractory disease, where subclonal heterogeneity and low shedding are most pronounced. Combining genomic variant tracking with methylation density, fragmentomic features, and, in the research setting, paired transcriptomic and proteomic characterization of metastatic tissue has revealed convergent mechanisms of progression, including antigen-presentation defects and immune evasion, that single-marker assays cannot resolve, and has simultaneously nominated actionable targets for the residual disease that conventional cytotoxics fail to eradicate [25,26].

### 2.4. Timing of Blood Collection and Quantitative Metrics

The postoperative landmark used in randomized trials has typically fallen between weeks 4 and 7, reflecting a practical balance: early enough to inform adjuvant decisions but late enough for surgical-related inflammatory cfDNA clearance. DYNAMIC drew blood at weeks 4 and 7, and GALAXY defined its primary MRD window at four weeks post-surgery [2,5]. Serial testing substantially outperforms any single time point, with the cumulative sensitivity across serial samples rising from approximately 60% at one landmark to over 85–95% in several prospective cohorts, and most ongoing trials now incorporate serial ctDNA monitoring throughout and after adjuvant therapy [29]. In a nationwide Danish cohort of 1001 patients with resected stage I–III CRC, more sensitive serial ctDNA quantification demonstrated a clinically meaningful lead time over conventional CEA- and imaging-based recurrence detection, underscoring the prognostic superiority of longitudinal molecular monitoring over single-timepoint assessment [30,31].

Beyond binary positivity, quantitative ctDNA metrics provide additional prognostic information on disease recurrence. Mutant tumor molecules per milliliter (MTM/mL) provides a continuous readout of residual tumor burden; in DYNAMIC-III, three-year recurrence-free survival fell sharply across ascending MTM/mL quartiles (78%, 63%, 36%, and 22%, respectively), a gradient no pathological variable reproduces [13]. In terms of ctDNA velocity, the doubling time of ctDNA burden between serial timepoints has been associated with markedly shorter RFS in post-hoc analyses, with a doubling time of ≤30 days portending particularly rapid progression [29]. Incorporating these continuous metrics as exploratory endpoints in future trials may recover prognostic signal that binary thresholds inevitably dilute, and may ultimately prove more useful than a simple positive/negative adjudication for treatment stratification.

## 3. Clinical Evidence by Disease Setting

### 3.1. Stage II Colon Cancer: The DYNAMIC Trial

The randomized DYNAMIC trial remains a pivotal reference for ctDNA-guided de-escalation in CRC. Tie et al. enrolled 455 patients with resected stage II colon cancer and randomly assigned them 2:1 to ctDNA-guided management (*n* = 302) or standard care (*n* = 153); blood was drawn at weeks 4 and 7, and ctDNA positivity at either timepoint triggered adjuvant chemotherapy at the treating clinician’s discretion [2]. The primary result was a halving of adjuvant chemotherapy use: 15.3% of patients in the ctDNA-guided arm received treatment, versus 27.9% in the standard arm (odds ratio 2.14, 95% CI 1.24–3.69, *p* = 0.002). Two-year recurrence-free survival was 93.5% versus 92.4%, meeting the pre-specified non-inferiority margin.

Five-year follow-up data, published in Nature Medicine in 2025, confirmed the durability of this finding [12]. Recurrence-free survival at five years was 88% in the ctDNA-guided group and 87% in the standard group (absolute difference 1.1%, 95% CI −5.8 to 8.0), with essentially identical overall survival (93.8% vs. 93.3%; HR 1.05, *p* = 0.887). Among ctDNA-positive patients treated with adjuvant chemotherapy, 87.5% achieved end-of-treatment ctDNA clearance, and sustained clearance predicted a 97% five-year RFS compared with 0% in those who remained ctDNA-positive post-treatment. This striking exploratory observation, while limited by small numbers and a post-hoc design, motivates prospective investigation of clearance as a therapeutic target.

DYNAMIC therefore establishes a clinically actionable principle: in stage II colon cancer, postoperative ctDNA can safely identify approximately 85% of patients who may forgo adjuvant chemotherapy, reducing systemic toxicity without compromising five-year survival. This study had some important limitations. This was a single-center-led phase II non-inferiority trial with a standard-arm chemotherapy rate of 28%, lower than that in many real-world practices, which limited the absolute reduction in chemotherapy achieved and constrained the power to detect small survival differences. The assay used (Signatera) also missed recurrences enriched in the lung and peritoneal sites, which are the known anatomical correlates of lower ctDNA shedding. Whether the favorable de-escalation experience observed under trial conditions generalizes to routine oncology practice remains an open question, given the single-center-led coordination, protocolized adherence, and lower-than-typical standard-arm treatment rate; the concordant real-world signal from the BESPOKE program provides partial reassurance, but confirmation in the ongoing multicenter randomized trials is required before the strategy can be regarded as broadly generalizable [17].

### 3.2. Stage III Colon Cancer: DYNAMIC-III and PEGASUS

The randomized phase 2/3 DYNAMIC-III trial extended the ctDNA-guided framework to locally advanced stage III colon cancer, testing both de-escalation and escalation strategies simultaneously within a 1002-patient trial [13]. The standard of care for stage III adjuvant therapy consisted of three to six months of oxaliplatin-based chemotherapy was established through the International Duration Evaluation of Adjuvant Chemotherapy (IDEA) collaboration, a pooled analysis of six randomized trials demonstrating that three months of CAPOX was effectively non-inferior in low-risk (T1–3N1) disease while six months of FOLFOX remained superior in high-risk (T4 or N2) tumors [32,33]. Against this backdrop, DYNAMIC-III set out to determine whether ctDNA could further individualize adjuvant intensity. ctDNA was measured 5–6 weeks post-resection; 72.5% of patients (*n* = 702) tested ctDNA-negative and were assigned to the de-escalation cohort, while 27.5% tested ctDNA-positive and were assigned to the escalation cohort. Both cohorts were randomized in a 1:1 ratio to ctDNA-guided or standard management.

In the ctDNA-negative de-escalation cohort, 90% of patients were de-escalated: oxaliplatin-containing chemotherapy was administered to 34.8% of patients in the guided arm versus 88.6% in the standard arm, respectively. The toxicity benefits were substantial, with grade ≥3 adverse events falling from 10.6% to 6.2% and hospitalizations from 13.2% to 8.5%. However, the primary endpoint of non-inferiority was not met: three-year RFS was 85.3% in the guided arm versus 88.1% in the standard arm (absolute difference −2.8%, 95% CI −8.0 to 2.3), which crossed the pre-specified −7.5% non-inferiority margin. A pre-planned subgroup analysis stratified by clinical risk suggested near-equivalent outcomes in the low-risk (T1–3N1) subgroup, where three-year RFS was 91.0% versus 93.2% (difference −2.2%, 97.5% lower CI −7.2%), with greater divergence in high-risk (T4 or N2) disease, implying that the clinical risk context remains important when applying ctDNA-guided de-escalation decisions. The divergence between the low-risk and high-risk strata is biologically coherent rather than incidental. Patients with T4 or N2 disease harbor a larger and more widely disseminated micrometastatic burden, so the absolute benefit withheld when oxaliplatin is omitted is correspondingly greater, consistent with the IDEA finding that six months of a doublet remains superior to abbreviated therapy in this stratum [32,33]. A negative ctDNA result is also less reassuring when the pre-test probability of residual disease is high, because the negative predictive value of any landmark assay declines as recurrence risk rises; the same false-negative rate that is tolerable in low-risk disease translates into a materially higher chance of undertreatment in T4 or N2 tumors. Compounding this, high-risk tumors more frequently recur at peritoneal and pulmonary sites, the anatomical compartments characterized by the lowest ctDNA shedding, so that ctDNA-negative status in this subgroup disproportionately reflects assay insensitivity rather than genuine absence of residual disease [13,30]. Taken together, these considerations argue that ctDNA-guided de-escalation should be applied most cautiously precisely where the baseline recurrence risk is highest, and that clinicopathological risk must continue to inform the decision alongside the molecular result.

The ctDNA-positive escalation cohort yielded more definitive negative results. Eighty-nine percent of guided-arm patients received intensified chemotherapy, with 56% receiving FOLFOXIRI chemotherapy. Despite this intensification, two-year RFS was 52% in the guided arm versus 61% in the standard arm (HR 1.11, 90% CI 0.83–1.48, *p* = 0.6). A sub-analysis comparing FOLFOXIRI to standard doublet (FOLFOX or CAPOX) within the guided arm found a three-year RFS of 47% versus 51%, directionally inferior with intensification. These results make it clear that delivering more cytotoxic drugs of the same mechanistic classes already failed by the primary tumor biology does not rescue MRD-positive patients [13].

The European investigator-initiated PEGASUS trial enrolled 135 patients with high-risk stage II and stage III colon cancer and assigned treatment based on tumor-naive liquid biopsy (Guardant Reveal LUNAR) ctDNA-positive patients who received three months of CAPOX, while ctDNA-negative patients received six months of capecitabine [18]. Confirmed prognostic performance was robust (two-year DFS 85.9% vs. 61.8%, HR 2.71; OS HR 3.11). An exploratory observation was that among patients remaining ctDNA-positive after CAPOX, approximately half achieved clearance on second-line FOLFIRI and remained recurrence-free, a finding that tentatively supports sequential biomarker-guided therapy as a hypothesis for prospective testing, although these numbers are small, uncontrolled, and potentially confounded by favorable disease biology.

### 3.3. The Post-Adjuvant Molecular Recurrence Setting: ALTAIR

The ALTAIR trial was the first completed randomized phase III study of a “treat-on-molecular-recurrence” (TOMR) strategy in any solid tumor type, and its results are the most consequential new data on ctDNA-guided CRC therapy as of 2026. The trial enrolled 243 patients across 46 Japanese centers who had undergone curative-intent resection of stage 0–IV CRC, completed standard adjuvant therapy, and subsequently developed Signatera-positive MRD with no radiological evidence of disease [14,15]. Patients were randomized 1:1 to six months of trifluridine/tipiracil (FTD/TPI; 35 mg/m^2^ twice daily for five of seven days per cycle) versus a matching placebo.

The primary endpoint was not met; the median disease-free survival was 9.30 months in the FTD/TPI arm versus 5.55 months for placebo (HR 0.79, 95% CI 0.60–1.05, *p* = 0.107). Grade ≥3 hematologic adverse events were substantially more frequent with FTD/TPI. Two secondary analyses warrant careful consideration of the following. First, a pre-specified subgroup analysis restricted to resected stage IV patients (*n* = 34 FTD/TPI vs. 32 placebo) showed a statistically significant DFS benefit (9.76 vs. 3.96 months; HR 0.53, 95% CI 0.32–0.87, *p* = 0.012), with this subgroup also demonstrating higher baseline MTM/mL (median 0.68 vs. 0.32, *p* = 0.024 across the full cohort), supporting the hypothesis that a higher ctDNA burden—and thus a larger target lesion burden—may be necessary for FTD/TPI to achieve a measurable DFS effect [14]. Second, a post-hoc analysis incorporating blinded central radiographic re-review reclassified some patients and yielded a statistically significant overall DFS benefit (HR 0.75, 95% CI 0.55–0.98, *p* = 0.041), suggesting that enrollment misclassification of radiological disease-free status may have diluted the intent-to-treat analysis.

Neither the subgroup finding nor the post-hoc reanalysis altered the primary conclusion: FTD/TPI monotherapy does not achieve a significant DFS benefit in unselected MRD-positive patients after adjuvant CRC therapy. Importance of ALTAIR lies less in what it proves than in what it teaches: the right drug matters as much as the right patient, and six months of a modestly active cytotoxic agent is not a sufficient strategy for eradicating chemotherapy-resistant micrometastatic clones.

### 3.4. Large-Scale Observational Validation: GALAXY and BESPOKE CRC

The GALAXY study within the CIRCULATE-Japan initiative provided the largest and most statistically robust validation of ctDNA as a prognostic biomarker in resected CRC. The initial cohort of 1039 stage II–III patients demonstrated a postoperative ctDNA positivity recurrence HR of 10.0 (95% CI 6.8–14.7, *p* < 0.0001), exceeding the prognostic strength of any prior biomarker in this disease [5]. The 23-month update, extending the cohort to 2240 patients across stages II–IV, confirmed DFS HR 11.99 and OS HR 9.68 (both *p* < 0.0001) [6]. A particularly important observation was the kinetics of ctDNA clearance: patients achieving sustained clearance (positive, then persistently negative) had a 24-month DFS of 89%, while those with only transient clearance (positive, then negative, then positive) had a 24-month DFS of 3.3%, essentially identical to persistently positive patients, a finding with direct implications for how clearance should be defined in therapeutic trials. The colorectal liver metastases subgroup (*n* = 190) further demonstrated that MRD-positive patients who received adjuvant chemotherapy had substantially improved 24-month DFS compared with observation (adjusted HR 0.07, *p* < 0.0001), with no detectable benefit in MRD-negative patients.

The BESPOKE CRC study enrolled 1792 patients across US academic and community oncology practices using the Signatera platform, providing real-world validation of ctDNA prognostic utility outside a trial context [17]. The final analysis presented at the ASCO Gastrointestinal Cancers Symposium 2025 confirmed MRD positivity rates of 7.5% in stage II and 28.3% in stage III disease, with a two-year DFS of 45.9% versus 91.8% in ctDNA-positive versus ctDNA-negative stage II patients (HR 11.23). Critically, ctDNA results influenced the management of 16% of patients; 60% of those changes involved de-escalation, 35% involved escalation, and 30% of ctDNA-detected recurrences were identified at a stage amenable to potentially curative metastasis-directed therapy. Patient-reported outcomes were notable: 96% of participants valued receiving ctDNA information, and 73% reported reduced anxiety about recurrence when the test result was negative.

A contrasting and cautionary result came from the COBRA trial (NRG-GI005), a randomized phase II/III study enrolling 635 patients with resected low-risk stage IIA colon cancer using the tumor-naive Guardant LUNAR assay [16]. Among the first 16 ctDNA-detected patients randomized, the phase II primary endpoint showed that ctDNA clearance at six months was not achieved, clearance occurred in 43% of patients under surveillance versus only 11% in the chemotherapy arm (*p* = 0.98), and the trial was closed early for futility. COBRA reinforces two critical lessons: conventional chemotherapy does not reliably induce ctDNA clearance, as measured by tumor-naive assay platforms, and clearance should not be assumed to be a surrogate therapeutic target until formally validated. These findings caution against designing future MRD trials with clearance as the sole primary endpoint until prospective surrogate validation is complete.

### 3.5. Rectal Cancer

In locally advanced rectal cancer, the treatment landscape has shifted dramatically toward total neoadjuvant therapy (TNT) and organ preservation strategies. The randomized OPRA trial demonstrated that TNT enabled organ preservation in approximately half of patients achieving clinical complete response, with five-year rectum-preservation rates of 77% for clinical complete responders [34]. ctDNA has two distinct potential roles in this evolving paradigm. First, serial ctDNA monitoring during TNT can track treatment response in near-real time. Studies using ultrasensitive assays have shown that ctDNA tumor fraction at the end of neoadjuvant treatment predicts sustained clinical complete response and can identify patients at imminent risk of regrowth during watch-and-wait surveillance, preceding regrowth by several months [35]. Second, post-resection or post-organ-preservation ctDNA can guide the intensity of subsequent surveillance, analogous to the colon cancer paradigm.

However, several caveats are specific to rectal cancer. Very low-level ctDNA positivity during and after neoadjuvant treatment may reflect treatment-associated inflammation rather than viable residual disease. Distinguishing true MRD from inflammatory cfDNA requires serial confirmation and quantitative metrics rather than single-sample interpretation. Additionally, the integration of ctDNA with rectal MRI response assessment, endoscopic evaluation, and CEA trending is not yet standardized, and no randomized trial has formally tested ctDNA-guided management in an organ-preservation setting. The ongoing interest in PD-1 blockade for dMMR rectal cancer, highlighted by Cercek et al.’s remarkable report of clinical complete response in all 49 patients completing dostarlimab therapy, raises the additional question of whether ctDNA might serve as a pharmacodynamic marker during immunotherapy [36].

### 3.6. Resected Oligometastatic CRC

After resection of colorectal liver metastases (CLMs) or lung metastases, recurrence rates exceed 70% at three years; however, the benefit of perioperative systemic chemotherapy has been contentious without clear randomized survival evidence. ctDNA is biologically well-suited for this setting because CLM sheds ctDNA proportionally to the viable metastatic burden, making the postoperative MRD window particularly informative. In the GALAXY CLM subgroup (*n* = 190), MRD-positive patients who received adjuvant chemotherapy demonstrated markedly improved 24-month DFS compared with MRD-positive patients under observation alone (adjusted HR 0.07, *p* < 0.0001), while MRD-negative patients showed no survival benefit from adjuvant treatment [6]. These observational findings cannot substitute for randomized evidence, but they generate a clinically coherent hypothesis: ctDNA can identify the subset of CLM patients who genuinely harbor residual disease and are therefore most likely to benefit from adjuvant chemotherapy, while sparing ctDNA-negative patients unnecessary treatment. The ALTAIR stage IV subgroup which is the only cohort in that trial showing a statistically significant DFS benefit from FTD/TPI, is also consistent with this framework. Prospective randomized trials using ctDNA to select CLM patients for adjuvant therapy are a high-priority gap in the evidence base.

## 4. The Prognostic-to-Predictive Gap: Mechanisms and Implications

**Figure 2:**
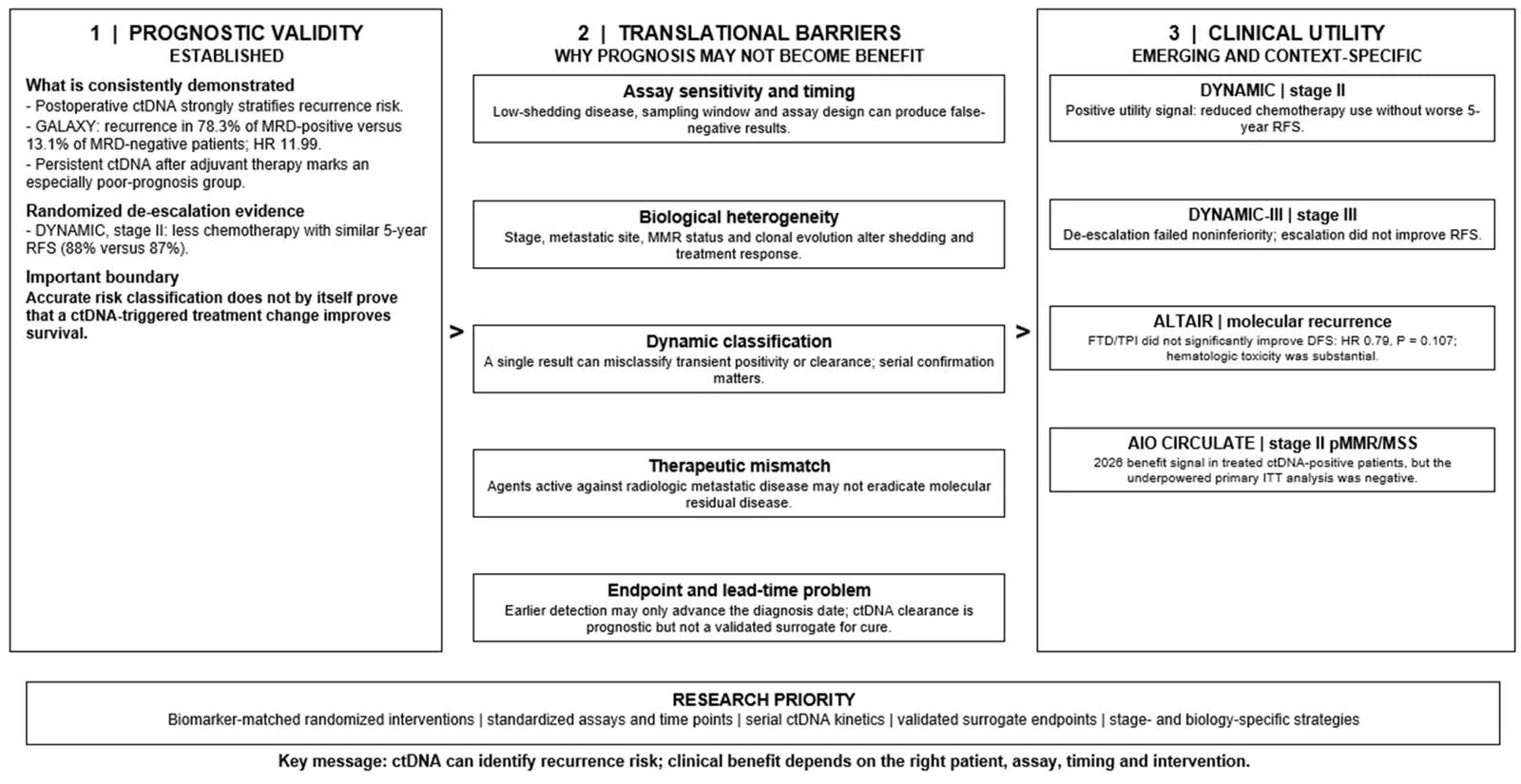
The prognostic-to-predictive gap in ctDNA-based molecular residual disease research. A conceptual diagram illustrating the distinction between clinical validity (prognostic utility: the ability to accurately stratify recurrence risk, as demonstrated by hazard ratios exceeding 10 across GALAXY, BESPOKE, and DYNAMIC) and clinical utility (predictive utility: the ability to improve patient outcomes when acted upon). The six mechanistic contributors to the gap are depicted as barriers between validated prognostic performance and unproven therapeutic benefit: (i) inappropriate escalation agents; (ii) assay false negatives in low-shedding anatomical recurrence sites (peritoneum and lung); (iii) transient versus biologically persistent ctDNA positivity; (iv) lead-time bias compressing the DFS advantage available for detection; (v) clonal evolution beyond the primary tumor panel; and (vi) unvalidated ctDNA clearance as a surrogate endpoint. Arrows indicate the research priorities required to close each mechanistic gap.

The central paradox of ctDNA in resected CRC is now well established, in which postoperative ctDNA predicts recurrence more accurately than any prior marker. However, no intervention triggered by ctDNA positivity has been shown to meaningfully reduce recurrence or improve survival in key escalation and TOMR settings. Understanding this requires disentangling several overlapping biological and methodological explanations. These mechanisms are conceptually illustrated in Figure 2.

### 4.1. The Validity–Utility Distinction

A prognostic biomarker stratifies patients by outcome, regardless of the treatment. A predictive biomarker identifies patients who are likely to respond to specific interventions. These properties are logically independent, and the field has conflated them too readily [37]. ctDNA has robust clinical validity and reliably indicates who will relapse. However, clinical utility requires that this knowledge leads to effective treatment decision-making. If no sufficiently active treatment exists for ctDNA-positive patients, prognostic accuracy is medically consequential for prognosis counseling and surveillance intensity, but not for adjuvant chemotherapy decisions. The DYNAMIC-III and ALTAIR results suggest that the treatments deployed were insufficiently active against the biology they were meant to target, not that the underlying concept of MRD-guided escalation was incorrect.

### 4.2. Mismatch Between Escalation Therapy and Tumor Biology

In DYNAMIC-III, escalation meant adding irinotecan to a fluoropyrimidine/oxaliplatin backbone (FOLFOXIRI), thereby delivering more of the same mechanistic drug classes that the primary tumor had already demonstrated it was capable of surviving from. ctDNA positivity after standard surgery may in fact enrich for biologically aggressive, inherently drug-resistant disease which is the very feature that makes ctDNA a powerful prognostic marker in identifying patients whose tumors shed sufficient viable cells to be detectable and may also identify tumors with intrinsic resistance to conventional cytotoxics [13]. Similarly, in ALTAIR, FTD/TPI is a third-line agent with response rates of 2–4% in platinum-refractory metastatic CRC; deploying it as the first escalation therapy in the adjuvant setting was unlikely to eradicate disease capable of surviving first-line oxaliplatin [14].

### 4.3. Assay False Negatives and Anatomically Low-Shedding Recurrences

ctDNA-based MRD detection has a non-trivial false-negative rate. At a single four-week timepoint, clinical sensitivity ranges from 40% to 80% depending on the assay and stage, rising substantially with serial testing but never reaching 100% [24]. Critically, false negatives are not distributed randomly across anatomical recurrence sites; peritoneal metastases and lung-only recurrences are characterized by substantially lower ctDNA shedding than liver metastases or locoregional recurrences. In DYNAMIC-III, 13.5% of the ctDNA-negative cohort (95/702) ultimately relapsed; among these, lung-only recurrences accounted for 39% and peritoneal for 34%, which demonstrated precisely the anatomical patterns that low-shedding tumor biology would predict [13]. This means that ctDNA-negative status, while highly reassuring, is not equivalent to cure, and de-escalation trials should not be interpreted as establishing the safety of abandoning standard imaging surveillance in ctDNA-negative patients. The anatomical heterogeneity of ctDNA detection is, in turn, a manifestation of the underlying biology of ctDNA release and clearance, which is neither uniform across tumors nor governed principally by mutational content. Quantitative shedding correlates most strongly with tumor size and proliferative capacity, such that highly proliferative tumors release more ctDNA per unit burden, whereas low-proliferation and stroma-rich histologies, including the secretory and consensus-molecular-subtype-3 phenotypes, shed comparatively little, and microsatellite-instable tumors tend to shed more [38]. Superimposed on these tumor-intrinsic determinants are compartmental barriers to hematogenous release; peritoneal deposits in particular are separated from the systemic circulation by the mesothelial plasma–peritoneal interface and therefore contribute disproportionately little DNA to plasma, which explains why peritoneal and pulmonary recurrences yielded the lowest postoperative detection rates, on the order of 20% and 19%, respectively, in a large nationwide cohort [30]. Differential clearance across organ microenvironments, driven by variation in the efficiency with which apoptotic and necrotic debris is processed at each site, further modulates the plasma signal independently of the true residual burden. These mechanisms collectively caution that a negative postoperative result should be interpreted in light of the expected shedding behavior of the anatomical and molecular disease context rather than as a uniform readout of cure.

### 4.4. Transient Positivity and Biological Noise

Not all ctDNA-positive results reflect established micrometastatic deposits in patients. Low-level variant signals can arise from clonal hematopoiesis, postsurgical inflammatory cfDNA release, or technical noise in samples close to the analytical detection threshold. The distinction between biologically persistent MRD and transient or technical positivity is clinically critical because treatment triggered by a false positive exposes a patient to chemotherapy toxicity without benefit [39]. GALAXY provided a sobering demonstration of this: patients who showed apparent ctDNA clearance (initially positive, then testing negative at the next timepoint) had a 24-month DFS of 3.3%, which is as poor as those who remained continuously positive, suggesting that a single negative confirmatory sample does not indicate genuine eradication [6]. Future trial designs should require at least two consecutive positive samples before triggering intervention, incorporate MTM/mL thresholds rather than binary cutoffs, and distinguish low-burden positivity from high-burden trajectories with distinct escalation strategies.

### 4.5. Lead-Time Bias and the Therapeutic Window Problem

The median interval between ctDNA detection and radiological recurrence, approximately 5–8 months across published series, defines the therapeutic window that TOMR strategies must exploit [19,20]. In ALTAIR, this window was consumed by six months of an insufficiently active drug, after which patients transitioned to radiological disease with limited subsequent treatment options. Consequently, TOMR designs must be powered not merely to show DFS improvement, a relatively modest bar, but to convert molecular recurrence into long-term disease control. To achieve this, the drug deployed must be capable of reducing the viable tumor burden by several logs rather than merely slowing its growth. A related statistical concern is that earlier detection compresses the time before the control arm progresses, reducing the DFS advantage available for detection, even when the intervention is effective. Moreover, the lead time is not a fixed interval but varies with the biology of the eventual recurrence. High-shedding hepatic and locoregional relapses are typically heralded by a longer molecular lead time, whereas low-shedding pulmonary and peritoneal recurrences may be detected only shortly before radiological progression or missed altogether, and histological subtypes with intrinsically low shedding compress the window further [30,38]. Consequently, the frequently cited interval of five to eight months represents an average across a heterogeneous population rather than a dependable therapeutic runway, and the relationship between earlier molecular detection and any genuine extension of the actionable treatment window remains to be established prospectively for each disease context.

### 4.6. ctDNA Clearance as an Unvalidated Surrogate Endpoint

The most frequently proposed solution for long-term survival follow-up in MRD trials is to use ctDNA clearance as a surrogate endpoint for DFS or OS. The appeal is obvious: if a drug clears ctDNA, it presumably eliminates residual disease and prevents recurrence; however, empirical evidence does not yet support this inference. The COBRA trial demonstrated that adjuvant chemotherapy in ctDNA-positive stage II patients did not increase ctDNA clearance at six months (43% vs. 11% in the chemotherapy and surveillance arms, respectively), even in a disease context where adjuvant fluoropyrimidines have a small survival benefit [16]. The FDA’s 2024 final guidance on ctDNA explicitly states that clearance has not been validated as a surrogate for OS in any solid tumor type [40]. Until a prospective randomized trial demonstrates that drugs achieving ctDNA clearance also improve DFS and that drugs failing to achieve clearance do not, clearance cannot be used as a primary endpoint in regulatory submissions or as the sole basis for treatment intensification decisions in clinical practice.

### 4.7. Clonal Evolution and Panel Design Limitations

Tumor-informed panels are designed based on somatic variants present in the primary tumor at the time of resection. However, distant metastases may harbor additional mutations acquired during clonal evolution, and adjuvant chemotherapy exerts a selection pressure that enriches for drug-resistant subclones not represented in the original panel [41]. Longitudinal ctDNA analyses during treatment have detected the emergence of acquired resistance mutations, including KRAS amplification, EGFR extracellular domain mutations, or TP53 co-mutations that are not trackable by panels built from the pre-treatment primary. This limitation argues for tumor-naive or hybrid assay approaches in the surveillance setting, where capturing clonal evolution may be as important as tracking established variants, and underscores the value of repeat ctDNA profiling during and after therapy rather than relying solely on the post-resection panel.

## 5. Clinical Implementation: Integration, Equity, and Guideline Landscape

### 5.1. Integration with Conventional Staging and Molecular Biomarkers

ctDNA results should augment, rather than replace, conventional staging and molecular profiling methods. In practice, the most clinically useful application is the combination of ctDNA with the existing risk stratification framework: a ctDNA-negative result in a patient with dMMR stage II colon cancer, already a population with minimal adjuvant chemotherapy benefit, provides strong additional support for observation; conversely, a ctDNA-positive result in a patient with pT3N0 MSS disease brings the recurrence risk estimate into the range typically associated with stage III, shifting the risk–benefit calculus decisively toward adjuvant treatment. The GALAXY dataset confirmed that integrating ctDNA with BRAF mutation status and MMR profiling outperforms any individual variable in risk stratification [5]. CEA and imaging remain complementary rather than redundant; a rising ctDNA concurrent with rising CEA carries greater specificity for genuine MRD than either marker alone, and cross-sectional imaging is still needed to characterize anatomical recurrence patterns and direct site-specific therapy once the disease becomes radiologically apparent. It follows that ctDNA is best regarded as one input within a multifactorial decision rather than an irreplaceable or free-standing determinant of adjuvant therapy. Its incremental weight relative to established indicators varies with context: a negative result adds most where the clinicopathological pre-test probability of residual disease is already low, and contributes least where high-risk features, mismatch-repair status, or anatomical patterns predictive of low shedding indicate that a negative signal may be unreliable. In resource-constrained settings in particular, validated stage- and biomarker-based risk stratification remains the backbone of decision-making, with ctDNA serving to refine rather than replace it.

### 5.2. Equity, Accessibility, and Assay Standardization

The adoption of ctDNA MRD testing in routine oncology practice faces substantial structural barriers that are systematically understated in clinical-trial reports. Tumor-informed assays require adequate archival FFPE tumor material (failure rates of 5–10% in real-world practice), specialized laboratory infrastructure, and 10–21 days of turnaround time, which is a logistical challenge in healthcare systems where adjuvant chemotherapy must be initiated within 8–12 weeks of surgery to be effective. Commercial pricing for Signatera in the United States falls between $3400 and $5000 per assay time point, with reimbursement coverage that remains inconsistent across insurance payers, and is unavailable in most healthcare systems globally. These economics raise equity concerns that are not theoretical, in which patients in underserved communities, rural settings, and lower-income countries, often those at the highest absolute risk of CRC, are least able to access technologies whose benefits currently accrue predominantly in affluent academic centers. These constraints reinforce that ctDNA testing, however informative, is not yet an indispensable component of standard adjuvant decision-making, and that equitable care cannot be made contingent on access to a costly assay of imperfect sensitivity; the demonstrable clinical value of ctDNA in defined scenarios must therefore be weighed against the imperative that established, universally available staging and risk-stratification tools continue to anchor management wherever the test is unavailable.

Assay standardization presents similar challenges. Signatera, Guardant LUNAR/Reveal, NeXT Personal, PROPHET, and methylation-based platforms each use distinct panel designs, variant filtering algorithms, and clinical cut-offs calibrated against different reference data sets. Cross-trial comparisons are therefore inherently imprecise: a positive Signatera result does not carry the same sensitivity or specificity as a positive Guardant LUNAR result in the same patient, and reporting thresholds differ [24]. The GUIDE.MRD consortium, established to benchmark competing assays head-to-head in prospective CRC cohorts, represents a critically important infrastructural investment. Until standardized analytical performance thresholds are established and independently validated, results from different assay platforms should not be considered interchangeable in clinical or research settings.

### 5.3. Current Guideline Positions

Despite compelling prognostic data, no major oncological society or regulatory body currently endorses routine ctDNA-guided modification of adjuvant treatment decisions outside of clinical trials (Table 3). The NCCN Colon Cancer guidelines (v2.2025) categorize postoperative ctDNA as a category 2A prognostic marker but explicitly state that it should not be used to guide adjuvant therapy decisions in standard practice, noting that “participation in clinical trials is encouraged.” [42] ASCO and the College of American Pathologists, in the most recent joint guideline document, found insufficient evidence to recommend ctDNA for MRD-guided treatment decisions and noted that ongoing trials are required before clinical utility can be established [39]. ESMO’s Precision Medicine Working Group similarly does not recommend ctDNA for MRD detection in the curative setting, while endorsing validated ctDNA platforms for RAS/BRAF/KRAS genotyping in advanced disease [34]. The US FDA’s 2024 final guidance on ctDNA in curative-intent drug development articulates a regulatory framework for ctDNA-based patient enrichment and response monitoring but confirms that ctDNA clearance is not a validated surrogate endpoint for OS in any solid tumor type [40].

## 6. Future Directions

### 6.1. Biomarker-Matched Escalation Strategies

**Figure 3:**
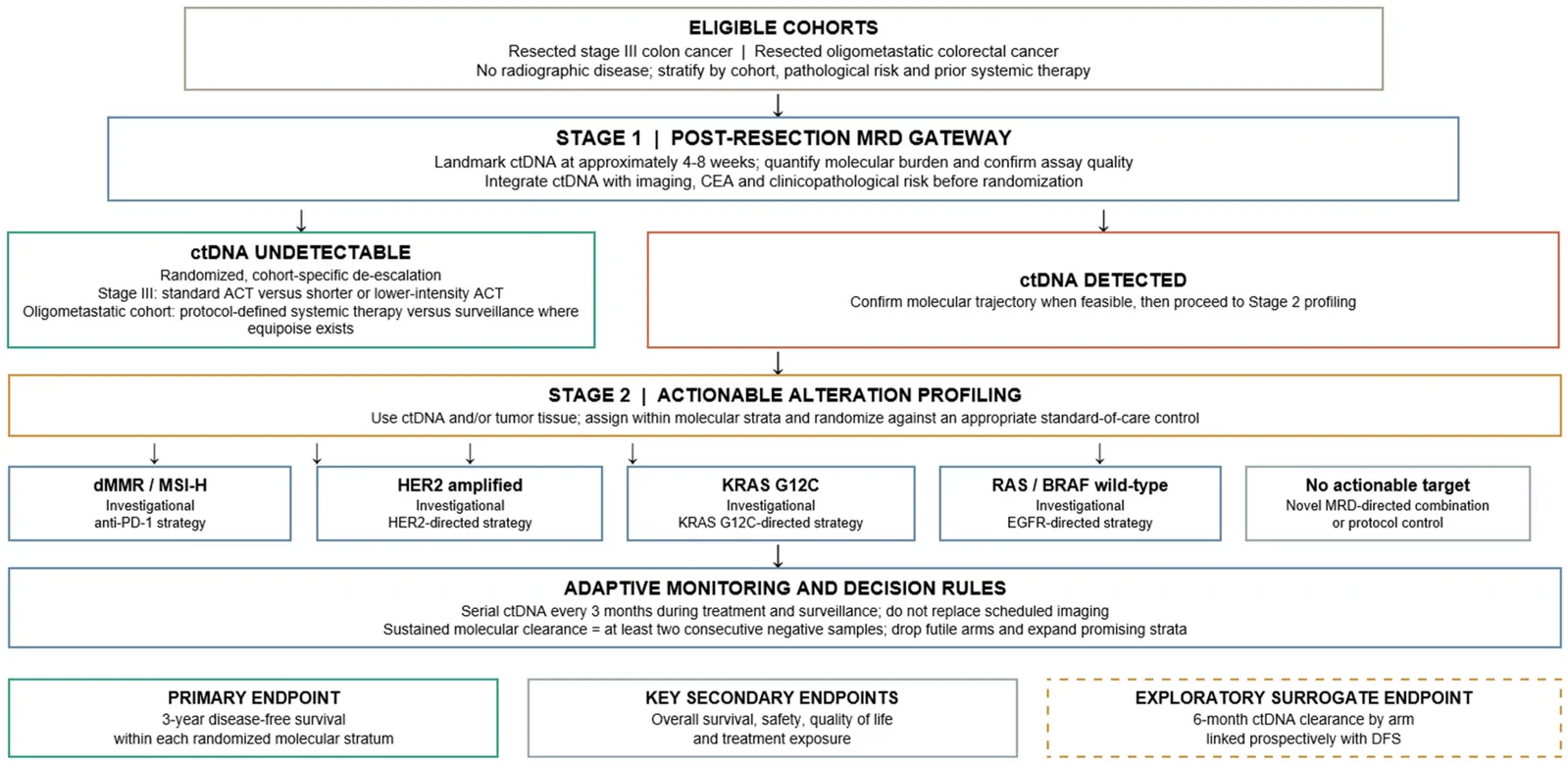
Proposed framework for a next-generation biomarker-matched adaptive MRD clinical trial in resected CRC. Schematic depicting a two-stage ctDNA-adaptive trial design applicable to stage III colon cancer and resected oligometastatic disease. Stage 1 (post-resection MRD window, 4–7 weeks): patients are stratified by quantitative ctDNA status (ctDNA-negative: low-burden de-escalation arm; ctDNA-positive: proceed to Stage 2 molecular characterization). Stage 2 (actionable alteration profiling from ctDNA or tumor): patients are matched to biomarker-directed escalation arms based on identified molecular targets (dMMR → anti-PD-1 therapy; HER2 amplification → HER2-directed therapy; KRAS G12C → KRAS G12C inhibitor; RAS/RAF wild-type → EGFR-directed therapy; no actionable target → novel combinations or observation). Serial ctDNA monitoring every 3 months serves as a pharmacodynamic marker throughout treatment, with sustained clearance defined as two consecutive negative results required before declaring MRD clearance. Primary endpoint: disease-free survival at 3 years; pre-specified co-primary endpoint (exploratory): ctDNA clearance rate at 6 months within each biomarker-matched arm (to facilitate surrogate endpoint validation). dMMR = deficient mismatch repair; EGFR = epidermal growth factor receptor; ctDNA neg = ctDNA-negative; ctDNA pos = ctDNA-positive.

The failure of empiric cytotoxic intensification in DYNAMIC-III and ALTAIR strongly argues that the next generation of ctDNA-positive escalation trials must be molecularly matched. Rather than asking only, “is it ctDNA-positive?”, future designs should interrogate the molecular content of the ctDNA signal to identify actionable targets [41]. A patient with ctDNA-detected HER2 amplification might receive pertuzumab plus trastuzumab in the adjuvant setting; dMMR ctDNA-positive disease could be enrolled in a pembrolizumab or nivolumab TOMR trial analogous to the landmark PD-1 blockade work in locally advanced rectal cancer [36]; KRAS G12C-positive ctDNA might trigger a sotorasib adjuvant pilot. This multi-drug, biomarker-matched TOMR framework mirrors the strategy that has transformed the treatment of metastatic CRC and is the most theoretically sound next step toward proven clinical utility in the MRD setting. The proposed biomarker-matched adaptive MRD trial framework is shown in Figure 3.

Among biomarker-matched strategies, the mismatch-repair-deficient subset occupies a special position because of the exceptional sensitivity of these tumors to immune checkpoint blockade, exemplified by the complete clinical responses observed with neoadjuvant dostarlimab in mismatch-repair-deficient rectal cancer, and ctDNA is well suited to serve as a pharmacodynamic readout of response during such therapy [36]. The more difficult problem is the microsatellite-stable, ctDNA-positive population, which constitutes the majority of molecular-residual-disease cases and has historically been regarded as immunologically cold. Two lines of evidence now motivate testing immunotherapy in this group: exome-scale analyses implicating antigen-presentation defects and active immune evasion as principal drivers of microsatellite-stable progression, which imply a targetable immune interaction, and the recognition that no approved therapy exists for these patients [25]. The EMPIRE platform trial (NSABP FC-13) directly addresses this gap, randomizing patients with microsatellite-stable stage II-III or resected oligometastatic stage IV colorectal cancer who remain ctDNA-positive after definitive therapy to cemiplimab alone or in combination with the anti-LAG-3 antibody fianlimab or a costimulatory bispecific agent, with ctDNA clearance at 12 weeks as the primary endpoint [43]. By adopting molecular clearance as an early efficacy signal within a randomized platform design, EMPIRE exemplifies the biomarker-matched, clearance-anchored approach advocated here, and its results will help clarify whether ctDNA clearance can serve as a surrogate for durable benefit in the immunotherapy setting.

### 6.2. Adaptive De-Escalation Trial Architecture

The strongest near-term evidence supports the de-escalation of treatment. The CIRCULATE-US (NRG-GI008) trial is enrolling up to 1912 patients with high-risk stage II and stage III resected colon cancer; ctDNA-negative patients are randomized to immediate versus observation-only (delayed) chemotherapy, while ctDNA-positive patients are randomized between standard doublet (FOLFOX/CAPOX) and intensified triplet (FOLFIRINOX) chemotherapy [42]. This trial architecture, in which de-escalation and escalation hypotheses are tested in parallel within the same trial, with ctDNA serving as a continuous adaptive biomarker rather than a one-time trigger, is the most rigorous design for definitively establishing ctDNA’s clinical utility in the colon cancer adjuvant setting. The companion CIRCULATE-Japan VEGA trial tests ctDNA-guided observation versus CAPOX in ctDNA-negative patients and will contribute crucial de-escalation safety data outside the Western clinical context.

### 6.3. Endpoint Innovation and Surrogate Validation

Three technical and regulatory advances could substantially accelerate this field. First, establishing ctDNA clearance as a validated surrogate endpoint in at least one defined clinical subgroup, analogous to pathological complete response in early-stage breast cancer, would enable smaller, faster TOMR trials without the multi-year event-driven survival follow-up currently required [37]. This validation requires a meta-analytic dataset demonstrating that the treatment effect on ctDNA clearance reliably predicts the treatment effect on OS across multiple independent trials, a dataset that does not yet exist for CRC. Second, incorporating quantitative ctDNA metrics (MTM/mL, ctDNA velocity, and clearance kinetics) as co-primary or ranked secondary endpoints in all future trials would build the cross-study dataset needed for eventual surrogate validation and would enable earlier pharmacodynamic proof-of-concept signals. Third, the GUIDE.MRD benchmarking consortium, assay standardization initiatives, and pre-competitive data-sharing arrangements are prerequisites for any cross-platform regulatory framework and deserve sustained international investment.

### 6.4. Rectal Cancer and Oligometastatic Settings as Priority Areas

Two disease settings should be prioritized in the next trial cycle. In rectal cancer, integrating ctDNA into organ-preservation protocols as a biomarker to guide re-treatment decisions during watch-and-wait, rather than purely as a surveillance adjunct, represents a biologically compelling application with no competing standards of care. In the oligometastatic CRC setting, ctDNA-selected adjuvant chemotherapy after CLM resection is the most immediately actionable hypothesis supported by existing observational data, and a randomized trial powered for DFS in this population would be both feasible and impactful. Both settings offer higher ctDNA shedding rates and shorter disease-free intervals than early-stage colon cancer, potentially broadening the therapeutic window and making the statistical power requirements for intervention trials more achievable.

## 7. Conclusions

The evidence on ctDNA-guided adjuvant and post-adjuvant therapy in resected CRC as of mid-2026 supports three clear conclusions and imposes three equally clear caveats for clinical practice: First, ctDNA-guided de-escalation in stage II colon cancer is a therapeutic application with demonstrated safety and maintained survival in a mature randomized dataset. Five-year results from DYNAMIC confirmed that approximately 85% of stage II patients were ctDNA-negative and safely spared adjuvant chemotherapy without compromising recurrence-free or overall survival. This has real clinical value in avoiding six months of fluoropyrimidine or oxaliplatin-based chemotherapy preventing neuropathy, gastrointestinal toxicity, cytopenias, and associated financial and quality-of-life burdens. De-escalation in ctDNA-negative stage III patients shows similar toxicity benefits but has not formally met non-inferiority criteria and therefore remains investigational.

Second, ctDNA-guided escalation and post-adjuvant monotherapy at molecular recurrence do not improve survival with the agents tested thus far. The intensification arm of the DYNAMIC-III and ALTAIR trials converged on the same conclusion through different experimental designs: knowing that a patient will relapse before it happens does not help them unless an effective treatment can prevent or meaningfully delay that relapse. The negative results do not refute the ctDNA concept; rather, they refute empirical cytotoxic intensification as a strategy for ctDNA-positive patients. Third, the mechanistic explanation for the prognostic-to-predictive gap is multifactorial and addressable, which includes wrong agents, assay false negatives in low-shedding anatomical recurrence sites, transient positivity masquerading as MRD, unvalidated surrogate endpoints, and clonal evolution beyond the primary tumor panel. These are not insurmountable problems but rather a research agenda.

The path from the current state of the field to proven clinical utility in escalation and TOMR settings runs through biomarker-matched drug selection, adaptive randomized trial designs, formal surrogate endpoint validation, and health equity, ensuring that innovations validated in academic trial settings reach the patients who most need them. The DYNAMIC results indicate that ctDNA is ready for clinical use in specific, well-defined scenarios. The ALTAIR and DYNAMIC-III results indicate that the more difficult task of converting prognostic accuracy into therapeutic impact has not yet been achieved. For the clinician at the bedside, the practical guidance is clear: use ctDNA to counsel patients and intensify surveillance where access permits, discuss results within shared decision-making frameworks, and route ctDNA-positive patients whenever possible to trials that might actually change what happens next.

## Figures and Tables

**Table 1:** Landmark randomized and prospective ctDNA trials in resected colorectal cancer. RFS = recurrence-free survival.

Trial (Year)	N	Stage/Setting	Design	Key Result	Clinical Implication
DYNAMIC [2,12] (2022; 5-yr 2025)	455	Stage II colon	Randomized phase II; ctDNA-guided vs. standard	Adjuvant chemo: 15.3% vs. 27.9% (*p* = 0.002); 5-yr RFS 88% vs. 87%; 5-yr OS 93.8% vs. 93.3%	De-escalation safe; spares 85% of patients chemo without survival compromise
DYNAMIC-III [13] (2025)	1002	Stage III colon	Randomized phase II/III; guided vs. standard	ctDNA-neg de-escalation: 3-yr RFS 85.3% vs. 88.1% (non-inferiority not met); ctDNA-pos escalation: 2-yr RFS 52% vs. 61% (HR 1.11)	Escalation with FOLFOXIRI fails; de-escalation reduces toxicity but non-inferiority was not met
ALTAIR [14,15] (2026)	243	Resected stage 0–IV; post-adj MRD+	Randomized phase III; FTD/TPI vs. placebo	Median DFS 9.30 vs. 5.55 months (HR 0.79, *p* = 0.107); benefit in resected stage IV (HR 0.53, *p* = 0.012)	Single-agent FTD/TPI fails overall; stage IV subset warrants further study
GALAXY (CIRCULATE-Japan) [5,6] (2023/2024)	2240	Stage II–IV (observational)	Prospective cohort; Signatera 4-wk landmark	4-wk ctDNA positivity: DFS HR 11.99, OS HR 9.68 (both *p* < 0.0001); sustained clearance: 24-mo DFS 89% vs. 3.3%	Strongest prognostic biomarker described in resected CRC; ctDNA clearance trajectory is critical
COBRA/NRG-GI005 [16] (2024)	635	Low-risk stage IIA	Randomized phase II/III; ctDNA-directed vs. surveillance	Chemo did not increase ctDNA clearance (43% surveillance vs. 11% chemo, *p* = 0.98); trial closed early	Clearance is not a reliable chemo-response surrogate; assay platform matters
BESPOKE CRC [17] (2025)	1166	Stage II–III (real-world)	Prospective US observational; Signatera	2-yr DFS 45.9% vs. 91.8% (ctDNA+ vs, stage II, HR 11.23); 16% of oncologists changed management	Confirms prognostic value; earlier recurrence detection enables metastasis-directed therapy in 30%
PEGASUS [18] (2023/2025)	135	High-risk stage II/stage III	Prospective; Guardant LUNAR; ctDNA-guided chemo selection	2-yr DFS 85.9% (ctDNA) vs. 61.8% (ctDNA+); serial testing post-CAPOX enables salvage FOLFIRI clearance	Sequential biomarker-guided therapy may achieve clearance after first-line failure
INTERCEPT (MD Anderson) [19,20] (2023)	1140+	Stage II–IV (surveillance)	Prospective real-world program; Signatera serial	NPV 98.1%; false-negative rate 1.3%; median ctDNA lead time 5.5 months before radiological recurrence	High NPV supports surveillance utility; long lead time enables intervention window

DFS = disease-free survival; HR = hazard ratio; FTD/TPI = trifluridine/tipiracil; NPV = negative predictive value; MRD = molecular residual disease; adj = adjuvant.

**Table 2:** Comparison of tumor-informed and tumor-naive ctDNA assay strategies for postoperative minimal residual disease detection.

Feature	Tumor-Informed (Bespoke)	Tumor-Naive (Agnostic)
Representative assays	Signatera (Natera); NeXT Personal (Personalis); PROPHET	Guardant Reveal/LUNAR; SPOT-MAS; xM assay (methylation + SNV hybrid); plasma WES tumor-agnostic (WES-TA/TAV16)
Tumor tissue required	Yes—FFPE required; failure in 5–10% (low purity or inadequate tissue)	No—plasma only
Assay design turnaround	10–21 days from tumor submission to clinical result	Typically 5–10 days (no upfront tumor sequencing)
Analytical sensitivity (LOD)	0.01% VAF; ultra-deep approaches reach 0.004–0.005%	0.1–0.25% VAF (fixed panels); methylation assays vary
Specificity for CHIP artifacts	High—variants are pre-matched to known tumor somatic mutations	Lower—fixed panels cannot exclude CHIP without paired tumor reference
Clinical trials validated in	DYNAMIC, DYNAMIC-III, GALAXY, ALTAIR, BESPOKE (Signatera)	COBRA, PEGASUS (Guardant); GALAXY subset (xM)
Detection at 4-week post-op	GALAXY tumor-informed: sensitivity 60–80% for recurrent cases	GALAXY xM: clinical sensitivity 61.1%, specificity 87.9% at landmark
Serial surveillance advantage	Cumulative sensitivity rises to 85–95% with serial testing	Improves but single-point sensitivity remains lower; better for screening contexts
Cost (approximate, US)	$3400–5000 per timepoint; reimbursement variable	$1800–3500 per timepoint; broader commercial availability
Capture of clonal evolution	Limited to variants in original tumor panel; may miss metastasis-specific subclones	Fixed panels can capture acquired resistance mutations not present in primary tumor
Best suited for	MRD detection at early low-burden timepoints (4–8 weeks post-op); high-specificity confirmatory testing	Broad surveillance programs; resource-constrained settings; tumor-tissue unavailable

FFPE = formalin-fixed, paraffin-embedded; VAF = variant allele fraction; CHIP = clonal hematopoiesis of indeterminate potential; LOD = limit of detection; SNV = single-nucleotide variant.

**Table 3:** Current guideline and regulatory positions on ctDNA-guided treatment modification in resected colorectal cancer.

Society/Body	Year	Evidence Position on ctDNA	Practice Recommendation
NCCN (Colon Cancer Guidelines)	2025	ctDNA is prognostic (recurrence risk factor) but not yet proven predictive; category 2A evidence for prognostic use	Do not use ctDNA to modify adjuvant therapy outside clinical trials; “participation in clinical trials is encouraged”
ASCO/CAP (Merker et al. [37])	2018	Insufficient evidence to recommend ctDNA for MRD-guided treatment; ongoing trials required for clinical utility determination	ctDNA should not replace standard pathological staging or standard-of-care adjuvant decisions; no recommendation for MRD-guided treatment
ESMO Precision Medicine Working Group (Pascual et al. [39])	2022	ctDNA is not recommended for MRD detection due to lack of demonstrated clinical utility; validated for genotyping in metastatic CRC	Recommend ctDNA testing only within prospective clinical trials for MRD purposes; endorsed for RAS/BRAF genotyping in advanced disease
NCI GI ctDNA Working Group (Rajdev et al. [42])	2025	ctDNA has clinical validity as prognostic marker; clinical utility for treatment decisions remains unproven; clearance is not a validated surrogate for OS/DFS	Prospective validation required before ctDNA serves as a trial primary endpoint; frameworks for patient enrichment and MRD monitoring in drug development provided
US FDA Final Guidance [40]	2024	ctDNA has potential roles in patient selection, MRD-based enrichment, response measurement, and exploratory early efficacy endpoints in curative-intent drug development	High NPV/sensitivity needed for de-escalation support; high PPV/specificity needed for escalation support; clearance not yet a validated surrogate; guideline is advisory, not approval-based

NCCN = National Comprehensive Cancer Network; ASCO = American Society of Clinical Oncology; CAP = College of American Pathologists; ESMO = European Society for Medical Oncology; NCI = National Cancer Institute; FDA = US Food and Drug Administration; OS = overall survival; DFS = disease-free survival.

## Data Availability

Data is contained within the article.
